# Examining Regional Differences of Dietary Inflammatory Index and Its Association with Depression and Depressive Symptoms in Korean Adults

**DOI:** 10.3390/ijerph17093205

**Published:** 2020-05-05

**Authors:** Dayeon Shin, Nitin Shivappa, James R. Hébert, Kyung Won Lee

**Affiliations:** 1 Department of Food and Nutrition, Inha University, Incheon 22212, Korea; dyshin@inha.ac.kr; 2 Cancer Prevention and Control Program, University of South Carolina, Columbia, SC 29208, USA; shivappa@email.sc.edu (N.S.); JHEBERT@mailbox.sc.edu (J.R.H.); 3 Department of Epidemiology and Biostatistics, Arnold School of Public Health, University of South Carolina, Columbia, SC 29208, USA; 4 Department of Nutrition, Connecting Health Innovations LLC, Columbia, SC 29201, USA; 5 Department of Food Science and Nutrition, Gwangju University, Gwangju 61743, Korea

**Keywords:** depression, depressive symptoms, dietary inflammatory index, Korea National Health and Nutrition Examination Survey (KNHANES)

## Abstract

The relationship between the dietary inflammatory index (DII^®^) and depression and depressive symptoms in South Korean adults remains unclear. This study aimed to examine the overall relationship between the DII and depression in South Korea and to evaluate the association between the DII and depressive symptoms and depression across regions among Korean adults aged ≥19 years. A total of 15,929 study participants were selected from the Korea National Health and Nutrition Examination Survey (KNHANES) 2014–2017. Energy-adjusted (E-DII) scores were calculated using 24-h dietary recall data. Depression and depressive symptoms were measured on the basis of the Korean version of the Patient Health Questionnaire 9-item scale, a doctor’s diagnosis of depression, and self-reported depressive symptom-related questionnaire. Overall, 4.2% of the participants had depression, with notable gender differences (i.e., 2.4% in men and 6.2% in women). Korean adults residing in the Capital area, Chungcheong-do and Jeju-do, and with diets in the highest tertile of the E-DII (most pro-inflammatory diet) had significantly increased odds of having depression and depressive symptoms compared with those in the lowest tertile of the E-DII (most anti-inflammatory diet) after controlling for covariates (adjusted odds ratio (AOR): 1.44, 95% confidence interval (CI): 1.04–1.99; AOR 2.97, 95% CI 1.36–6.52; AOR 4.06, 95% CI 1.56–10.53, respectively). No association between the E-DII and depression/depressive symptoms was found in other regions of South Korea. A pro-inflammatory diet is associated with greater odds of depression and depressive symptoms, with distinct regional differences. The present study provides evidence regarding existing regional differences in the association of the E-DII with depression and depressive symptoms.

## 1. Introduction

Depression is a major health concern affecting more than 300 million people worldwide, which accounts for 4.4% of the world’s population [1]. One of the significant factors that can contribute to the occurrence of depression is nutrition. “Healthy” dietary patterns characterized by high intakes of whole grains, vegetables, fruits, fish, nuts, and seeds are associated with lower risk of depression among United States (U.S.) women [2]. In a randomized controlled study, participants in the Mediterranean-style diet group with fish oil supplementation had significantly greater improvement in depression risk than the social group provided with snacks such as biscuits, cheese, dips, tea/coffee, and water/juice [3]. This may be partially due to the fact that people with depression are more likely to engage in unhealthy lifestyle behaviors [4]. Furthermore, low socioeconomic status (SES) plays a key role in psychiatric disorders, and SES inequalities are partly explained by regional discrepancies among the population. For example, mental health was found to be associated with the level of regional social deprivation in Wales, England [5].

The relationship between diet and depression can be partly explained by inflammation. Depression has been associated with increased levels of interleukin-6 (IL-6) [6,7], tumor necrosis factor-alpha (TNF-α) [8], and C-reactive protein (CRP) [8]. For instance, previous findings reported increased circulating levels of cytokines in the cerebrospinal fluid of depressed patients, and a positive correlation was found between serum IL-1β and the severity of depression [9]. Results of the analyses from the 2009–2010 National Health and Nutrition Examination Survey (NHANES) indicate that study participants with depression had higher CRP concentrations; that is, 47.01% of the study population with depression had CRP levels of ≥3.0 mg/L, and 29.06% had CRP levels of ≥5.0 mg/L [10]. This finding partially explains the relationships between inflammation and the higher risk of comorbidities associated with depression [11]. Moreover, inflammatory cytokines may predict depression. People who had higher serum levels of IL-6 at age nine had 1.55 higher odds of depression when they reach the age of 18 years as reported in the Avon Longitudinal Study of Parents and Children in England [12]. When individuals are exposed to chronic inflammation, this will cause changes in neurotransmitter metabolism, which will lead to psychiatric disorders, specifically depression [13]. Peripheral cytokines can access the brain and activate inflammatory networks of the local central nervous system to produce alterations in neurotransmitter function and may lead to the development of depression [14].

One of the main factors modulating inflammation is diet. To quantify the inflammatory potential of diet, the dietary inflammatory index (DII^®^) was developed on the basis of the extant medical literature published through 2010 [15] and validated with IL-6, high-sensitivity CRP (hs-CRP), and TNF-α in Western countries [16,17,18,19]. We previously validated the DII in Korean adults using hs-CRP [20]. The relationship between the DII and depression or depressive symptom risk has been explored in the U.S. [21,22,23] and European countries [24,25,26,27]; however, to the best of our knowledge, no such studies have evaluated the relationship between the DII and depression in Korean adults. Given the influence of the regional differences with respect to mental health, data on the status of depression and depressive symptoms in South Korea are limited. Hence, this study aimed to achieve the following objectives: (1) to examine the regional differences in the DII in South Korea, and (2) to evaluate the association of the DII and depressive symptoms and depression stratified by regions using a representative sample of Korean adults using the Korea National Health and Nutrition Examination Survey (KNHANES).

## 2. Methods

### 2.1. Study Population

**Figure 1:**
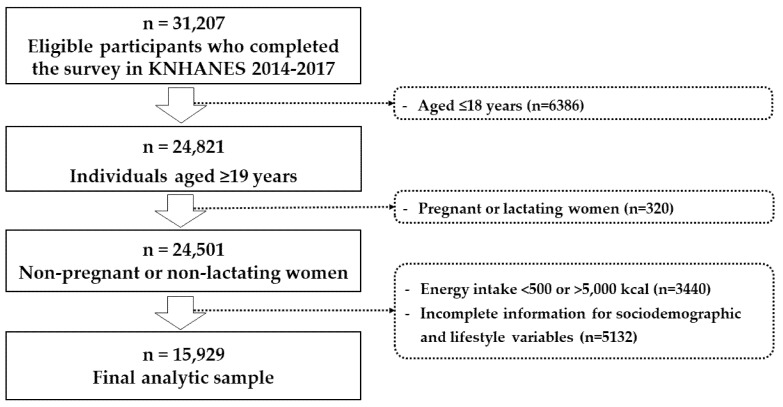
Flow chart of study participants.

The study included Korean adults aged ≥19 years who participated in the KNHANES 2014–2017. A total of 31,207 participants completed the survey in KNHANES 2014–2017. After excluding participants aged <19 years (n = 6386), pregnant or lactating women (n = 320), those with energy intake of <500 or >5000 kcal/day (n = 3440), and those with incomplete sociodemographic and lifestyle data (n = 5132), a total of 15,929 (7201 men and 8728 women) were included in the final analysis (Figure 1). The institutional review board (IRB) of the by the Korea Centers for Disease Control and Prevention (KCDC) reviews and approves the KNHANES survey (approval no. 2013-12EXP-03-5C). Informed consent was obtained from all participants when the surveys were conducted. IRB approval was not required because the KNHANES is a publicly available dataset.

### 2.2. Dietary Inflammatory Index (DII^®^)

The DII was developed by researchers at the University of South Carolina. Details on the development and validation of the DII have been published previously [15,17]. In brief, the literature (approximately 2000 articles) published between 1950 and 2010 was reviewed in terms of the relationship between various micronutrients, macronutrients, and whole food items (termed food parameters) and inflammation to obtain the inflammatory effect scores of the food parameters. At the same time, a global database, which contains the means and standard deviations of intake of food parameters from 11 populations around the world, was created [15]. For this study, the KNHANES dietary data, based on a single 24-h dietary recall, were used to calculate the DII. The original DII includes 45 food parameters; however, only 22 of the 45 parameters were available and used for the calculation of the DII in this study. The following 22 DII food parameters were included: carbohydrates, protein, fat; vitamin A, vitamin B_1_, vitamin B_2_, vitamin B_3_ (niacin), vitamin C, iron, saturated fatty acids, monounsaturated fatty acids, polyunsaturated fatty acids, omega-3 and omega-6 polyunsaturated fatty acids, dietary fiber, cholesterol, β-carotene, garlic, ginger, onion, pepper, and tea. The world mean value of each food parameter was subtracted from the actual; i.e., reported intake value of each food parameter and then divided by the world standard deviation to create a z-score. Next, the z-scores were converted to proportions (with values from 0 to 1), which were then centered on zero by doubling the value and subtracting 1 (i.e., with values ranging from −1 to +1). This value was then multiplied by the inflammatory effect score of each food parameter. These were then summarized across all food parameters to obtain the overall DII score. More positive scores indicate a higher intake of a pro-inflammatory diet; negative scores indicate a higher intake of an anti-inflammatory diet [15]. In this study, the energy-adjusted DII (E-DII^TM^) scores were calculated per 1000 calories by converting all nutrients from KNHANES. A global database with dietary exposures expressed per 1000 kcal/day was then used to calculate the E-DII scores.

### 2.3. Assessment of Depression and Depressive Symptoms

Using the self-reported Korean version of the Patient Health Questionnaire 9-item (PHQ-9) scale, depression was defined as having a PHQ score of ≥10 [28] or a doctor’s diagnosis of having depression. PHQ-9 was measured in KNHANES 2014 and 2016 only. For depressive symptoms, the study participants were asked the following question: “Has your life been disrupted by feelings of hopelessness or sadness for more than two weeks within the past year?” Participants who responded “yes” to that question were classified as having depressive symptoms. 

### 2.4. Definition of Regions

**Figure 2:**
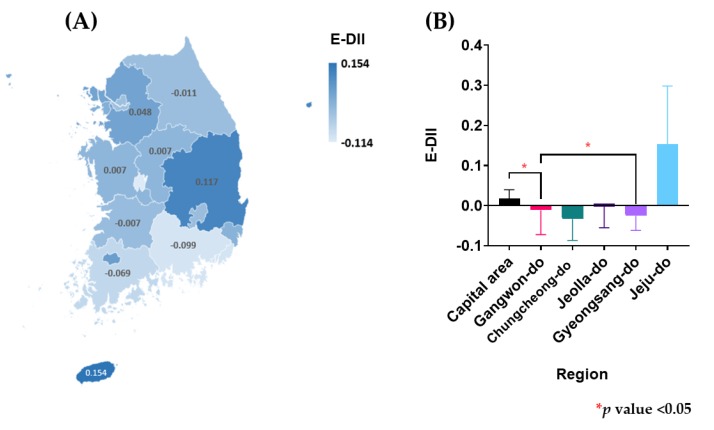
Mean energy-ajusted dietary inflammatory index (E-DII) score by (**A**) 16 cities and provinces and (**B**) 6 regions.

The regions were divided into the following groups (see Figure 2): (1) 16 cities and provinces including Seoul, Busan, Daegu, Incheon, Gwangju, Daejeon, Ulsan, Gyeonggi-do, Gangwon-do, Chungcheongbuk-do, Chungcheongnam-do, Jeollabuk-do, Jeollanam-do, Gyeongsangbuk-do, Gyeongsangnam-do, and Jeju-do, and (2) six regions including the Capital area (Seoul, Incheon, and Gyeonggi-do), Gangwon-do, Chungcheong-do (Deajeon, Chungcheongbuk-do, and Chungcheongnam-do), Jeolla-do (Gwangju, Jeollabuk-do, and Jeollanam-do), Gyeongsang-do (Busan, Daegu, Ulsan, Gyeongsangbuk-do, and Gyeongsangnam-do), and Jeju-do.

### 2.5. Statistical Analyses

The descriptive statistics of sociodemographic factors; lifestyle factors such as alcohol consumption, smoking status, and physical activity; body mass index (BMI) and regions were computed and stratified by tertiles of the E-DII. Chi-square tests were used to examine the differences in categorical variables by tertiles of the E-DII score. Mean E-DII was calculated by 16 cities and provinces and six regions. T-test was performed to examine the differences of mean E-DII between the two regions. Multivariable logistic regression analyses were used to calculate the adjusted odds ratios (AORs) and 95% confidence intervals (CIs) for the association between the tertiles of the E-DII and depression stratified by 6 regions after controlling for covariates including age, gender, education, alcohol consumption, smoking habits, physical activity, and BMI. The sample weights of the participants were constructed to represent the Korean population by accounting for the complex survey design, survey non-response, and post-stratification. All statistical analyses were conducted using SAS^®^ version 9.4 (SAS Institute, Cary, NC, USA). Statistical significance was declared at *p* value <0.05.

## 3. Results

Table 1 presents the sociodemographic and lifestyle variables by E-DII tertiles. Gender, age, household income, education, employment status, drinking habits, smoking status, and BMI significantly differed across the E-DII tertiles (*p* value <0.001). The study participants in tertile 3 (most pro-inflammatory diet) were more likely to be men (60.3%), were aged 30–49 years (41.9%), had the highest quartile of household income level (30.8%), had a higher education level (58.4%), were blue-collar workers (44.2%), consumed alcohol 1–4 times/month (40.9%), were nonsmokers (70.7%), and had a normal weight (39.7%). E-DII tertile 3 had the highest number of smokers (29.3%) compared to tertile 1 (15.2%).

The mean E-DII score in 16 cities and provinces and six regions in South Korea are indicated in Figure 2. Variability of estimates in the regional subgroups was high in KNHANES, particularly in regions with a small number of subjects (n = 300) in Jeju-do. Daejeon showed the lowest E-DII score (indicating the most anti-inflammatory diet) (−0.114), while Jeju-do showed the highest E-DII score (0.154). When divided into six regions in South Korea, Jeju-do showed the highest E-DII, while Chungcheong-do and Gyeongsang-do showed the lowest E-DII. Participants who are residing in the Capital area had a significantly higher E-DII scores than in those participants who are residing in Gangwon-do and Gyeongsang-do, respectively (0.018 vs. −0.011 and −0.026; *p* values <0.05). 

Table 2 presents the distributions of sociodemographic and lifestyle factors and E-DII scores based on the status of depression. Out of 15,929 adults, 4.2% had depression, with a notable difference that disfavored women (6.2%) compared with men (2.4%). Gender, age, household income, education, employment status, and drinking status all significantly differed by status of depression (all *p* values <0.05). Korean adults with depression were more likely to be women (69.6%), aged 50−64 years (34.4%), had the lowest quartile of household income (27.1%), had 7–12 years of education (46.7%), were unemployed (56.5%), and were nondrinkers (50.2%).

The mean and standard errors of the mean of the E-DII were calculated for study participants with or without depression stratified according to the six regions in South Korea as indicated in Table 3. There were no significant differences in E-DII score by status of depression. Smoking status, physical activity, BMI, regions, and E-DII score did not differ by status of depression.

The relationships of the E-DII with the risk of depression and depressive symptoms by six regions in South Korea are shown in Table 4. The study participants who lived in the Capital area and were in the highest E-DII tertile (who had the most pro-inflammatory diet) had significantly increased odds of having depression and depressive symptoms compared with those in the lowest E-DII tertile, after controlling for covariates (Adjusted odds ratio (AOR) 1.44, 95% CI: 1.04–1.99). Those adults who resided in Chungcheong-do and Jeju-do had significantly increased odds of having depression and depressive symptoms (AOR 2.97, 95% CI 1.36–6.52; AOR 4.06, 95% CI 1.56–10.53, respectively). Those who lived in Gangwon-do in the mid-tertile of E-DII had significantly higher odds of having depressing and depressive symptoms (AOR 3.64, 95% CI 1.40–9.48). However, no significant association was observed between E-DII and depression and depressive symptoms in the other regions, Jeolla-do and Gyeongsang-do.

## 4. Discussion

The present study found that the E-DII score among Korean adults differed by cities and provinces of South Korea. Korean adults who resided in the Capital area including Seoul, Incheon, and Gyeonggi-do, Chungcheong-do, and Jeju-do consumed pro-inflammatory diets were more likely to have depression and depressive symptoms. However, no association was found between E-DII score and depression and depressive symptoms in Jeolla-do and Gyeongsang-do. To the best of our knowledge, this is the first study to consider the regional differences when evaluating the association between DII and depression and depressive symptoms. 

Using NHANES data, U.S. women with depressive symptoms demonstrated a higher DII than those without depressive symptoms [29]. In addition, in the Australian Longitudinal Study on Women’s Health, women who were in the lowest quartile of the DII had approximately 20% reduction in developing depression compared with those who were in the highest quartile of the DII [30]. In parallel with these findings, each standard deviation increase in the DII score was associated with 66% increased odds for recurrent depressive symptoms in the Whitehall II Study, which included a large cohort of British men and women [26]. Supporting this finding, Irish adults with a pro-inflammatory diet were associated with 70% increased odds of depressive symptoms, 60% higher odds of anxiety, and 38% lower odds of well-being compared with those with anti-inflammatory diets [27]. These study findings support that the pro-inflammatory diet is positively associated with the risk of depression and depressive symptoms, which is consistent with our findings on the positive association of the E-DII with depression and depressive symptoms among those who live in the Capital area, Chungcheong-do and Jeju-do. 

Genetic factors play a key role in the etiology of depression [31,32]. IL-6 promoter’s single nucleotide polymorphisms rs1800795 contributed to the increased risk of inflammation in individuals with lower socioeconomic status through β-adrenergic activation of the cyclic AMP (cAMP)/protein kinase A (PKA) pathway of the erythroid transcription factor (GATA-1) [33]. As genetic polymorphisms interact with the dietary inflammation–depression relationship, future studies are warranted to evaluate the effects of the interactions between genetic variants and dietary inflammation on the effect of depression.

In the present study, we evaluated the regional differences of the E-DII across the six regions in South Korea. Korean adults in the Capital area had a significantly higher E-DII than those who live in Gangwon-do and Gyeongsang-do. This finding indicates that people who live in the Capital area are more likely to demonstrate a pro-inflammatory diet than those who live outside of the Capital area. Wirth et al. reported that a higher DII was associated with lower diet quality [34], and diet quality is influenced by socioeconomic status [35,36]. This implies that the DII is influenced by socioeconomic status such as income inequalities that exist by region and may be due, in part, to regional differences in rurality. Kim and Jeong [37] reported that income inequality in South Korea was due to the differences as demonstrated by the relatively very high income in the Seoul Metropolitan Area, which is the most developed region of the nation.

This study has several strengths. It relied on a nationally representative sample of Korean adults using KNHANES. In addition, we were able to evaluate the regional differences of the E-DII in South Korea and determined whether the relationship between the E-DII and depression and depressive symptoms differed across region using the Korean national sample. In addition, using a large sample size, we controlled for numerous confounders in the analysis. Although the study has many strengths, it also has several limitations. The E-DII score was calculated using a single 24-h recall, and this may not reflect an individual’s usual dietary intake. The E-DII score was calculated based on only 22 out of 45 food parameters. However, we previously validated the E-DII in relation to high-sensitivity CRP in Korean adults using the 2015 KNHANES that also used single 24-h recall data [20]. Moreover, there are imbalances in terms of the numbers of individuals across regions. Inevitably, this will result in differences in the statistical power across sampling units. Due to the cross-sectional study design of the KNHANES, the cause-effect relationship between the inflammatory potential of diet and depression and depressive symptoms cannot be drawn. 

In conclusion, E-DII scores differed across six regions in South Korea and appeared to play a key role in the developing depression and depressive symptoms among individuals living in the Capital area, Chungcheong-do and Jeju-do. The present study provided evidence regarding the existence of regional disparities in different levels of dietary inflammation and differences in the association of the E-DII with depression and depressive symptoms.

## Figures and Tables

**Table 1:** Distributions of sociodemographic and lifestyle factors by tertiles of energy-adjusted dietary inflammatory index (E-DII).

	E-DII							
	Tertile 1 (n = 5309)		Tertile 2 (n = 5310)		Tertile 3 (n = 5310)		Total	
	n	Wt’d %	n	Wt’d %	n	Wt’d %	n	*p* Value ^1^
Gender								
Men	1998	43.5	2405	52.6	2798	60.3	7201	<0.0001
Women	3311	56.5	2905	47.4	2512	39.7	8728	
Age (year)								
19–29	315	10.0	559	16.4	1075	29.4	1949	<0.0001
30–49	1766	39.3	1928	41.3	2033	41.9	5727	
50–64	1899	34.0	1582	27.7	1113	17.7	4594	
≥65	1329	16.7	1241	14.5	1089	10.9	3659	
Household income								
Low	819	12.3	891	12.7	1026	14.7	2736	<0.0001
Middle low	1247	21.4	1318	22.9	1348	25.2	3913	
Middle high	1511	30.1	1508	31.3	1490	29.3	4509	
High	1732	36.2	1593	33.1	1446	30.8	4771	
Education								
≤Elementary school	849	11.2	920	11.3	891	9.9	2660	<0.0001
Middle/high school	2253	40.4	2078	36.7	1834	31.7	6165	
≥College	2207	48.4	2312	52.1	2585	58.4	7104	
Employment status (n = 15,906)								
Blue-collar Worker ^2^	1940	40.8	1989	42.8	2057	44.2	5986	<0.0001
White-collar Worker ^3^	1157	21.2	1298	23.9	1299	22.9	3754	
Unemployed	2208	37.9	2015	33.3	1943	32.8	6166	
Drinking status								
Nondrinker	2379	40.6	2165	37.0	1782	29.0	6326	<0.0001
1–4 times/month	1924	39.3	1915	38.4	1959	40.9	5798	
≥2 times/week	1006	20.1	1230	24.7	1569	30.0	3805	
Smoking status								
Nonsmoker	4669	84.8	4387	78.6	3961	70.7	13,017	<0.0001
Current smoker	640	15.2	923	21.4	1349	29.3	2912	
Physical activity ^4^								
No	2662	46.5	2847	48.8	2807	47.9	8316	0.16
Yes	2647	53.5	2463	51.2	2503	52.1	7613	
BMI (kg/m^2^)								
<18.5	166	3.4	218	4.3	260	5.1	644	0.0002
18.5–22.9	2153	40.3	2017	38.3	2094	39.7	6264	
23–24.9	1315	24.5	1244	22.9	1158	21.5	3717	
≥25	1675	31.7	1831	34.5	1798	33.7	5304	
Region								
Capital area	2728	54.1	2754	56.0	2741	56.1	8223	0.36
Gangwon-do	185	2.7	219	3.1	179	2.6	583	
Chungcheong-do	584	9.3	592	9.5	561	8.5	1737	
Jella-do	485	8.5	448	8.0	495	8.5	1428	
Gyeongsang-do	1237	24.7	1192	22.5	1229	23.3	3658	
Jeju-do	90	0.8	105	0.9	105	1.0	300	

^1^ Chi-square test. ^2^ Blue-collar workers were craft and related trades workers, drivers, plant and machine operators, assemblers, elementary occupation workers. ^3^ White-collar workers were chief executives, senior officials, legislators, managers, professionals, and technicians. ^4^ Physical activity was defined as meeting minimum criterion of the World Health Organization’s (2010) global physical activity recommendation: 150 min of moderate-intensity physical activity, 75 min of vigorous-intensity physical activity, or an equivalent volume of moderate- to vigorous-intensity physical activity (i.e., 600 METs minutes/week); Wt’d %: weighted percentage.

**Table 2:** Distributions of sociodemographic, lifestyle factor, and energy-adjusted dietary inflammatory index (E-DII) scores based on the status of depression.

	No Depression (n = 15,177; 95.8%)		Depression (n = 752; 4.2%)		
	n	Wt’d %	n	Wt’d %	*p* Value ^1^
Gender					
Men	7024	53.6	177	30.4	<0.0001
Women	8153	46.4	575	69.6	
Age (year)					
19–29	1891	19.5	58	13.7	<0.0001
30–49	5544	41.4	183	31.1	
50–64	4319	25.6	275	34.4	
≥65	3423	13.6	236	20.9	
Household income					
Low	2501	12.7	235	27.1	<0.0001
Middle low	3714	23.1	199	26.8	
Middle high	4361	30.6	148	21.0	
High	4601	33.6	170	25.1	
Education					
≤Elementary school	2443	10.3	217	19.7	<0.0001
Middle/high school	5832	35.5	333	46.7	
≥College	6902	54.1	202	33.6	
Employment status					
Blue-collar worker ^2^	5838	43.5	148	25.2	<0.0001
White-collar worker ^3^	3610	22.9	144	18.3	
Unemployed	5706	33.6	460	56.5	
Drinking status					
Nondrinker	5903	34.5	423	50.2	<0.0001
1–4 times/month	5590	40.0	208	30.5	
≥2 times/week	3684	25.5	121	19.3	
Smoking status					
Nonsmoker	12,390	77.5	627	78.9	0.46
Current smoker	2787	22.5	125	21.1	
Physical activity ^4^					
No	7899	47.7	417	49.1	0.53
Yes	7278	52.3	335	50.9	
BMI (kg/m^2^)					
<18.5	611	4.3	33	5.7	0.11
18.5–22.9	5988	39.5	276	36.8	
23–24.9	3553	23.0	164	20.8	
≥25	5025	33.2	279	36.8	
Region					
Capital area	7850	55.5	373	55.2	0.62
Gangwon-do	551	2.8	32	3.3	
Chungcheong-do	1644	9.1	93	8.6	
Jella-do	1371	8.4	57	6.8	
Gyeongsang-do	3477	23.4	181	25.0	
Jeju-do	284	0.9	16	1.2	
E-DII					
Tertile 1	5058	30.2	251	31.5	0.80
Tertile 2	5054	33.1	256	32.8	
Tertile 3	5065	36.7	245	35.7	

^1^ Chi-square test. ^2^ Blue-collar workers were craft and related trades workers, drivers, plant and machine operators, assemblers, elementary occupation workers. ^3^ White-collar workers were chief executives, senior officials, legislators, managers, professionals, and technicians. ^4^ Physical activity was defined as meeting the minimum criterion of the World Health Organization’s (2010) global physical activity recommendation: 150 min of moderate-intensity physical activity, 75 min of vigorous-intensity physical activity, or an equivalent volume of moderate- to vigorous-intensity physical activity (i.e., 600 METs minutes/week); Wt’d %: weighted percentage.

**Table 3:** Mean energy-adjusted dietary inflammatory index (E-DII) scores between study participants with or without depression.

Region	No Depression (n = 15,177; 95.8%)	Depression (n = 752; 4.2%)	*p* Value ^1^
Capital area ^2^ (n = 8223)	0.021 ± 0.021^1^	−0.051 ± 0.083	0.38
Gangwon-do (n = 583)	0.005 ± 0.060	−0.325 ± 0.272	0.26
Chungcheong-do (n = 1737)	−0.041 ± 0.053	0.147 ± 0.158	0.22
Jeolla-do (n = 1428)	0.001 ± 0.054	−0.085 ± 0.235	0.73
Gyeongsang-do (n = 3658)	−0.028 ± 0.037	0.027 ± 0.153	0.72
Jeju-do (n = 300)	0.144 ± 0.151	0.328 + 0.344	0.66

Data are presented as mean ± standard error of the mean. ^1^ *p* value based on t-test statistics. ^2^ Capital area includes Seoul, Incheon, and Gyeonggi-do.

**Table 4:** Associations of energy-adjusted dietary inflammatory index (E-DII) scores with the risk of depression and depressive symptoms by six regions in Korea.

Region	E-DII		
	Tertile 1	Tertile 2	Tertile 3
Capital area (n = 8223)	1.00 (Ref.)	1.22 (0.89–1.68)	1.44 (1.04–1.99) *
Gangwon-do (n = 583)	1.00 (Ref.)	3.64 (1.40–9.48) *	1.57 (0.57–4.33)
Chungcheong-do (n = 1737)	1.00 (Ref.)	1.92 (0.97–3.80)	2.97 (1.36–6.52) *
Jeolla-do (n = 1428)	1.00 (Ref.)	0.87 (0.52–1.47)	1.60 (0.88–2.90)
Gyeongsang-do (n = 3658)	1.00 (Ref.)	1.05 (0.73–1.51)	0.96 (0.63–1.47)
Jeju-do (n = 300)	1.00 (Ref.)	3.61 (0.93–13.99)	4.06 (1.56–10.53) *

Data are presented as adjusted odds ratios and 95% confidence intervals. Tertile 1 was set as a reference point. All models were adjusted for age, gender, education, occupation, alcohol consumption, smoking status, physical activity, and BMI. * *p* value < 0.05.
